# Calcium and magnesium in drinking water and risk of myocardial infarction and stroke—a population-based cohort study

**DOI:** 10.1093/ajcn/nqac186

**Published:** 2022-07-11

**Authors:** Emilie Helte, Melle Säve-Söderbergh, Susanna C Larsson, Agneta Åkesson

**Affiliations:** Unit of Cardiovascular and Nutritional Epidemiology, Institute of Environmental Medicine, Karolinska Institutet, Stockholm, Sweden; Unit of Cardiovascular and Nutritional Epidemiology, Institute of Environmental Medicine, Karolinska Institutet, Stockholm, Sweden; Science Division, Swedish Food Agency, Uppsala, Sweden; Unit of Cardiovascular and Nutritional Epidemiology, Institute of Environmental Medicine, Karolinska Institutet, Stockholm, Sweden; Unit of Medical Epidemiology, Department of Surgical Sciences, Uppsala University, Uppsala, Sweden; Unit of Cardiovascular and Nutritional Epidemiology, Institute of Environmental Medicine, Karolinska Institutet, Stockholm, Sweden

**Keywords:** calcium, magnesium, drinking water, myocardial infarction, stroke, cohort, population-based

## Abstract

**Background:**

The implication of calcium and magnesium in drinking water for cardiovascular disease is unclear.

**Objectives:**

To assess the association of the concentration of calcium and magnesium in drinking water with incidence of myocardial infarction and stroke, accounting for dietary mineral intake.

**Methods:**

We linked drinking water monitoring data to residential information of 26,733 women from the population-based Swedish Mammography Cohort, who completed a 96-item FFQ at baseline. Drinking water was categorized into low (magnesium <10 mg/L and calcium <50 mg/L) or high (magnesium ≥10 mg/L or calcium ≥50 mg/L) mineral concentration. Incident cases of myocardial infarction and stroke types were ascertained 1998–2019 using the National Patient Register.

**Results:**

The mean ± SD concentration of calcium and magnesium in drinking water was 29 ± 7 mg/L and 5 ± 1 mg/L in the low-exposed area and 52 ± 20 mg/L and 10 ± 3 mg/L in the high-exposed area, respectively. During 16 years of follow-up, we ascertained 2023, 2279, and 452 cases of myocardial infarction, ischemic stroke, and hemorrhagic stroke, respectively. High drinking water calcium and magnesium was associated with lower risk of ischemic and hemorrhagic stroke HRs of 0.87 (95% CI: 0.80, 0.95) and 0.78 (95% CI: 0.65, 0.95), whereas the HR for myocardial infarction was 0.93 (95% CI: 0.85, 1.02). In separate analyses, only drinking water magnesium, not calcium, remained associated with ischemic stroke (HR: 0.69; 95% CI: 0.54, 0.88).

**Conclusions:**

Drinking water with a high concentration of calcium and magnesium, particularly magnesium, may lower the risk of stroke in postmenopausal women.

## Introduction

Drinking water is our most important foodstuff, indispensable for human health. With climate change, our access to safe drinking water will become compromised, and by 2025, as much half of the world's population is expected to live in water-stressed areas (1). This will call for adaptations in the current drinking water production and increase the need of finding alternatives to conventional drinking water sources. Installation of filters and use of desalinated sea water are two increasingly popular examples of this. Both methods do, however, have the side effect of lowering the concentration of essential nutrients, such as calcium and magnesium in the drinking water. At present, the knowledge of the overall population health consequences of consuming drinking water with a low mineral content is unclear.

Drinking water with a high concentration of calcium and magnesium (also called hard water) has been repeatedly associated with a lower risk of cardiovascular mortality in ecological (2) and case-control studies (3–8). Yet, only few cohort studies have been published in this area (9–12), and of those, only 1 study (12) could confirm the protective association. In 2008, a systematic review and meta-analysis (13) concluded an inverse association with cardiovascular mortality for drinking water magnesium, but not calcium, which was later confirmed in an updated meta-analysis on magnesium alone (14). Although there are numerous studies on minerals and drinking water in relation to cardiovascular mortality, mainly death from myocardial infarction, the epidemiologic evidence on the association with stroke is very limited. To our knowledge, only 2 studies—1 small cohort study (9) and 1 case-control study (7)—with conflicting results have been published. Given that drinking water is our outermost consumed food and that cardiovascular diseases stand for the largest proportion of the global burden of disease, further investigation of this potential relation is warranted.

To corroborate the previous indications of a protective relation of drinking water calcium and magnesium with cardiovascular disease, we assessed the association of these minerals in drinking water with incidence of myocardial infarction and stroke in a prospective population-based cohort of 26,733 postmenopausal women, with low bottled water consumption (15, 16). We gathered detailed information on residential history, diet—including both dietary and supplemental calcium and magnesium, cardiovascular risk factors, and socioeconomic factors.

## Methods

### Study population

We used data from the Swedish Mammography Cohort (SMC), a population-based prospective cohort and part of the Swedish Infrastructure for Medical Population–based Life-course and Environmental Research (SIMPLER; www.simpler4health.se), with the aim of studying associations of diet and lifestyle with morbidity and mortality. The SMC was initiated in 1987–1990, when all women in Västmanland and Uppsala counties, born 1914–1948 (*n* = 90,303), were invited to participate in the study (74% response rate). In 1997, an extended questionnaire, that also included a 96-item FFQ, was sent out to all women still alive in the study area ( *n* = 56,030) to update information (70% response rate) on diet and lifestyle. This questionnaire was used as baseline for the present investigation. After excluding those with incorrect or missing personal identification number or a diagnosis of cancer or cardiovascular disease before baseline, 34,995 women remained in the study for further exposure assessment. The study was approved by the Regional Ethical Review Board in Stockholm, Sweden.

### Exposure assessment

**FIGURE 1:**
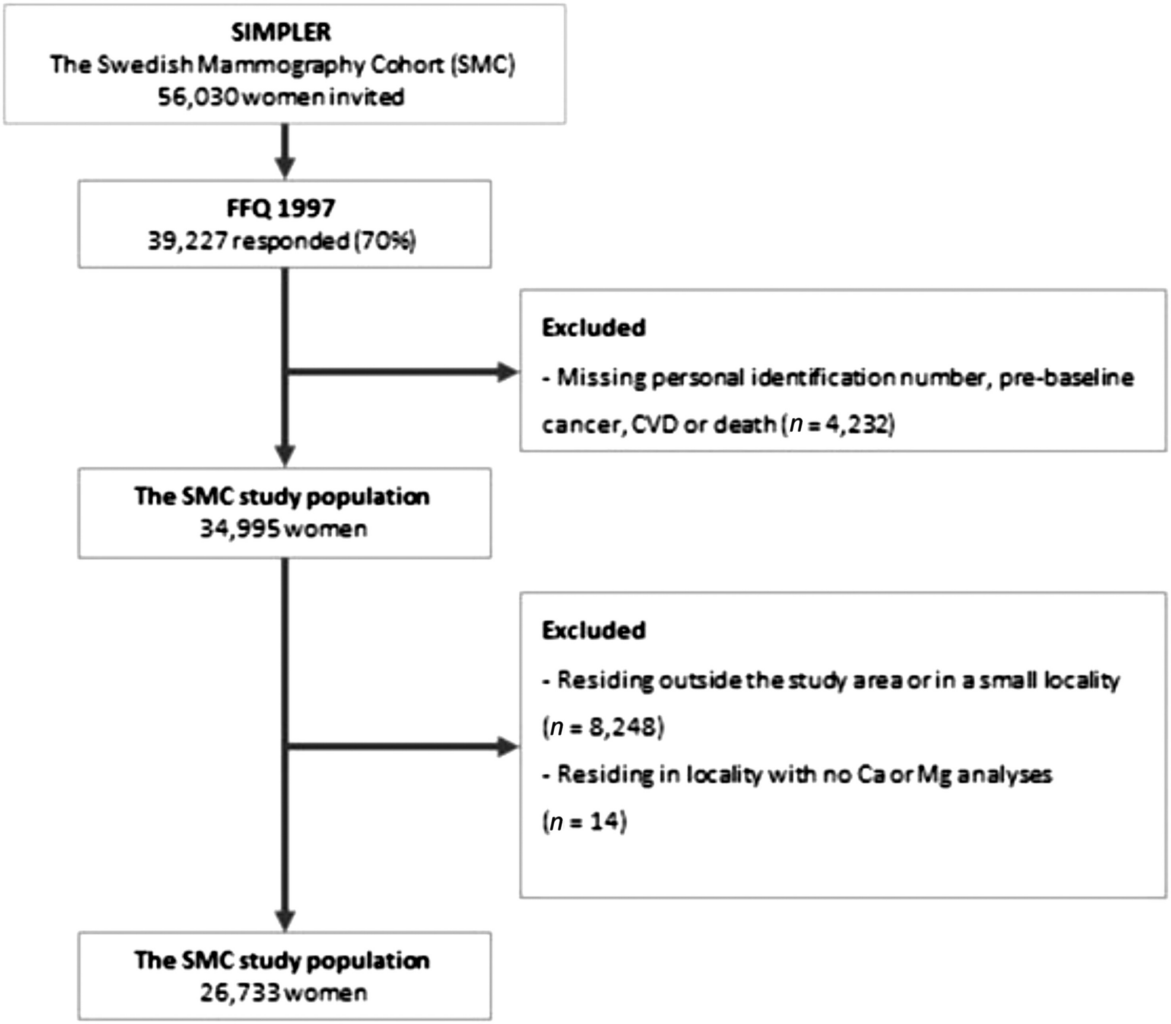
Flowchart of the Swedish Mammography Cohort study population. CVD, cardiovascular disease.

To reduce the risk of exposure misclassification and unmeasured confounding, only participants served by public drinking water were included in the study. First, we collected data on residential history (years 1997–2018) from the National Register for Regional Divisions Based on Real Estate (Statistics Sweden) and included everyone who was living in a locality (coherent and densely populated area) with ≥1000 inhabitants within the study area (*n* = 26,747) at baseline (1997). By restricting to participants living in localities, we minimized the risk of wrongfully including those using private wells. Thereafter, we mapped all drinking water treatment plants in the study area and collected monitoring data on concentrations of calcium and magnesium in drinking water from the Swedish Water analytical reports and Vattentäktsarkivet, Geological Survey of Sweden. If no monitoring data were available, or the drinking water treatment plants or source supplying the locality had changed during follow-up, we excluded the women living in these localities ( *n* = 14). This resulted in a final study population of 26,733 women (**Figure 1**).

We then calculated the average concentration of calcium and magnesium in drinking water for each locality at baseline. In cases when localities were served by >1 treatment plant, we used the average concentrations. To obtain yearly individual exposure information, starting from baseline in 1997 until end of follow-up in 2019, we matched each participant's residential history with the locality-specific exposure information. Finally, we categorized participants into low (<50 mg/L calcium and <10 mg/L magnesium) or high (≥50 mg/L calcium or ≥10 mg/L magnesium) exposure. The cutoff points were chosen based on a priori knowledge of the distribution of the calcium and magnesium concentration in public drinking water in the study area, using the median concentration of each mineral as the breaking point. To study the impact of calcium and magnesium separately, we also separated participants into tertiles of the exposure distribution for each of the minerals.

### Covariates

We collected information on education and household income from the Longitudinal Integrated Database for Health Insurance and Labor Market Studies (Statistics Sweden). Area-level income information, which we used as an indicator of potential contextual confounding, was obtained from Statistics Sweden, using demographic statistical areas (DeSO) (17). Data on diabetes came from the national diabetes and patient registers and from self-reports. Information on smoking habits, BMI, cholesterol levels, family history of cardiovascular disease, and physical activity came from the questionnaire completed at baseline in 1997. From the validated FFQ, we obtained data on alcohol consumption, dietary intake of calcium and magnesium, and use of calcium and/or magnesium supplements (18).

### Outcome assessment

We obtained information on incident cases of myocardial infarction (including out-of-hospital deaths in myocardial infarction, confirmed by autopsy) and stroke through a linkage of the cohort to the Swedish National Patient Register and the Cause of Death Register (National Board of Health and Welfare). The primary outcomes were myocardial infarction, ischemic stroke, and hemorrhagic stroke, whereas intracerebral hemorrhage, subarachnoid hemorrhage, and an extended definition of myocardial infarction were secondary outcomes. The following International Classification of Diseases, 10th revision codes were used to identify cases: I21 (myocardial infarction), I63–I64 (ischemic stroke), I60 (subarachnoid hemorrhage), I61 (intracerebral hemorrhage), and I60–I61 (hemorrhagic stroke). Moreover, we considered a broader definition of myocardial infarction, in which we also included women who had undergone revascularization procedures (percutaneous coronary intervention and coronary artery bypass grafting) as cases. These women were identified using the repair codes FNA-H and FNW.

### Statistical analysis

We used Cox proportional hazards regression with attained age as time scale to assess associations of drinking water calcium and magnesium with myocardial infarction and stroke, presented as HRs and 95% CIs. For each outcome-specific analysis, we calculated person time from 1 January 1998 until the date of diagnosis or repair, death, move from baseline locality, or end of follow-up on 31 December 2019, whichever occurred first.

For the main analysis where calcium and magnesium were assessed together, we built 4 different models: *1*) adjusted for age (as time scale); *2*) model 1 further adjusted for education, household income, smoking status, BMI, cholesterol, diabetes, physical activity, alcohol intake, and family history of cardiovascular disease; *3*) model 2 also adjusted for dietary intake of calcium and magnesium and use of calcium and/or magnesium supplements; and *4*) model 2 further adjusted for the median income in the area of residence. In the analyses where calcium and magnesium were assessed separately, all models were also adjusted for drinking water calcium or magnesium (depending on which mineral was analyzed). Because diet is the main source of calcium and magnesium, we also performed analyses stratified by the median dietary intake of each mineral. Last, to further explore any potential differences between drinking water calcium and magnesium in the association with myocardial infarction and stroke, we also performed analyses with drinking water minerals classified according to *1*) calcium <50 mg/L and magnesium <10 mg/L as reference, *2*) calcium >50 mg/L and magnesium <10 mg/L, and *3*) calcium <50 mg/L and magnesium ≥10 mg/L.

Missing information on covariates (<2% for all variables, except physical activity 10%) was handled using a missing indicator category for categorical variables and for continuous variables by replacing missing values with the median. The proportional hazard assumption was tested using Schoenfeld's residuals, and no indications of nonproportional hazards were observed. All tests were 2-sided, and the significance level was set at 0.05. The statistical software used was STATA/SE version 16.0 (StataCorp).

## Results

The mean ± SD concentration of calcium and magnesium in drinking water was 29 ± 7 mg/L and 5 ± 1 mg/L in the low-exposed area and 52 ± 20 mg/L and 10 ± 3 mg/L in the high-exposed area, respectively (**Supplemental Figures 1** and **2**). Age-standardized baseline characteristics of the study population are presented in **Table 1**. Corresponding descriptive information by tertiles of calcium and magnesium is presented in **Supplemental Tables 1** and **2**. The women in the high-exposed area were more likely to have a university education and had a slightly higher household income than the women in the low-exposed area, but apart from that, no major differences across exposure categories were observed.

**TABLE 1:** Baseline age-standardized main characteristics of the study population (*n* = 26,733) by drinking water calcium and magnesium exposure^1^

	Ca/Mg exposure	
Characteristic	Low Ca/Mg (Ca <50 mg/L and Mg <10 mg/L)(mean ± SD: 29 ± 7; 5 ± 1 mg/L)	High Ca/Mg (Ca ≥50 mg/L or Mg ≥10 mg/L)(mean ± SD: 52 ± 20; 10 ± 3 mg/L)
Number of participants	14,592	12,141
Age, mean ± SD, y	62 ± 9	62 ± 10
Education, y, %		
≤9	46	35
10–11	30	28
≥12	24	38
Household income, 1000 SEK/y, mean ± SD	231 ± 139	244 ± 197
Median area-level income, 1000 SEK^2^/y, mean ± SD	239 ± 37	254 ± 42
Smoking status, %		
Never	53	52
Former, <10 cigarettes/d	11	12
Former ≥10 cigarettes/d	11	12
Current <10 cigarettes/d	10	11
Current ≥10 cigarettes/d	14	12
BMI, kg/cm^2^, %	25.1 ± 3.9	24.7 ± 3.9
High cholesterol, %	7	9
Prevalent diabetes, %	4	4
Family history of cardiovascular disease, %	17	16
Physical activity, %		
Walk/bike ≥40 min/d	36	39
Exercise ≥1 h/wk	82	81
Alcohol consumption, %		
Never drinker	13	10
Former drinker	4	4
Drinker, ≤3 glasses/wk	54	52
Drinker, 3–7 glasses/wk	21	24
Drinker, ≥7 glasses/wk	8	10
Calcium intake, mean ± SD, mg/d	1049 ± 301	1046 ± 302
Magnesium intake, mean ± SD, mg/d	319 ± 43	320 ± 44
Calcium supplements, %	1	1
Magnesium supplements, %	4	3

1 Ca, calcium; Mg, magnesium.

2 1000 SEK = 100 EUR or 121 USD (exchange rate February 2021).

Over an average follow-up of 16 years (450,000 person-years), we ascertained 2023 cases of myocardial infarction and 2744 cases of stroke, of which 2279 were ischemic strokes, 349 were intracerebral hemorrhages, and 116 were subarachnoid hemorrhages. When extending the definition of myocardial infarction to also include women who had undergone revascularization procedures, we ascertained 258 additional cases.

Minerals in drinking water was associated with a multivariable adjusted HR of 0.93 (95% CI: 0.85, 1.02) for myocardial infarction, comparing drinking water with a high concentration with a low concentration (**Table 2**). The results were similar for the extended definition of myocardial infarction (**Supplemental Table 3**). The corresponding HRs for ischemic and hemorrhagic stroke were 0.87 (95% CI: 0.80, 0.95) and 0.78 (95% CI: 0.65, 0.95), respectively (Table 2). When separating hemorrhagic stroke into intracerebral hemorrhage and subarachnoid hemorrhage, the associations remained inverse for subarachnoid hemorrhage (HR: 0.67; 95% CI: 0.45, 0.99) and were slightly attenuated for intracerebral hemorrhage (HR: 0.83; 95% CI: 0.67, 1.03) (**Table 3**). Further adjusting the models for dietary and supplemental intake of calcium and magnesium or adjusting for area-level income had little or no impact on the results (**Supplemental Tables 4–6**).

**TABLE 2:** Hazard ratios of myocardial infarction, ischemic stroke, and hemorrhagic stroke by low and high drinking water calcium and magnesium exposure in 26,733 women of the Swedish Mammography Cohort^1^

			Age-standardized incidence rate/100,000 person years (95% CI)	HR (95% CI)	
Drinking water calcium and magnesium exposure	Cases, *n*	Person-years, *n*		Age-adjusted model^2^	Multivariable-adjusted model^3^
Myocardial infarction					
Ca/Mg <50/10 mg/L	1163	248,693	532 (492, 554)	1.00	1.00
Ca/Mg ≥50/10 mg/L	860	206,671	473 (441, 505)	0.89 (0.81, 0.97)	0.93 (0.85, 1.02)
Ischemic stroke					
Ca/Mg <50/10 mg/L	1326	247,877	604 (571, 639)	1.00	1.00
Ca/Mg ≥50/10 mg/L	953	206,462	530 (497, 565)	0.85 (0.79, 0.93)	0.87 (0.80, 0.95)
Hemorrhagic stroke					
Ca/Mg <50/10 mg/L	274	253,631	113 (99, 127)	1.00	1.00
Ca/Mg ≥50/10 mg/L	178	210,718	92 (79, 106)	0.79 (0.65, 0.95)	0.78 (0.65, 0.95)

1 Low calcium and magnesium is defined as <50 mg/L calcium and <10 mg/L magnesium, and high calcium and magnesium is defined as ≥50 mg/L calcium or ≥10 mg/L magnesium. Ca, calcium; Mg, magnesium.

2 Adjusted for age (as time scale).

3 Adjusted for age (as time scale), level of education (<9, 9, 10–11, 11–12, >12 y), household income (quartiles), smoking status (never, former <10 cigarettes/d, former ≥10 cigarettes/d, current <10 cigarettes/d, current ≥10 cigarettes/d), BMI (<20, 20–24.9, 25–29.9, and 30 kg/cm^2^), high cholesterol (yes/no), diabetes (yes/no), family history of cardiovascular disease (yes/no), physical activity (walk or bike ≥40 min/wk: yes/no; exercise ≥1 h/wk: yes/no), and alcohol intake (never drinker, former drinker, current drinker <3 glasses/wk, current drinker 3–7 glasses/wk, current drinker > 7 glasses/wk).

**TABLE 3:** Hazard ratios hemorrhagic stroke types by low and high drinking water calcium and magnesium exposure in 26,733 women of the Swedish Mammography Cohort^1^

			Age-standardized incidence rate/100,000 person years (95% CI)	HR (95% CI)	
Drinking water calcium and magnesium exposure	Cases, n	Person-years, *n*		Age-adjusted model^2^	Multivariable adjusted model^3^
Intracerebral hemorrhage					
Ca/Mg <50/10 mg/L	207	254,014	87 (75, 100)	1.00	1.00
Ca/Mg ≥50/10 mg/L	142	210,903	75 (62, 87)	0.83 (0.67, 1.03)	0.83 (0.67, 1.03)
Subarachnoid hemorrhage					
Ca/Mg <50/10 mg/L	75	254,473	28 (22, 35)	1.00	1.00
Ca/Mg ≥50/10 mg/L	41	211,190	20 (14, 26)	0.67 (0.45, 0.97)	0.67 (0.45, 0.99)

1 Low calcium and magnesium is defined as <50 mg/L calcium and <10 mg/L magnesium, and high calcium and magnesium is defined as ≥50 mg/L calcium or ≥10 mg/L magnesium. Ca, calcium; Mg, magnesium.

2 Adjusted for age (as time scale).

3 Adjusted for age (as time scale), level of education (<9, 9, 10–11, 11–12, >12 y), household income (quartiles), smoking status (never, former <10 cigarettes/d, former ≥10 cigarettes/d, current <10 cigarettes/d, current ≥10 cigarettes/d), BMI (<20, 20–24.9, 25–29.9, and 30 kg/cm^2^), high cholesterol (yes/no), diabetes (yes/no), family history of cardiovascular disease (yes/no), physical activity (walk or bike ≥40 min/wk: yes/no; exercise ≥1 h/wk: yes/no), and alcohol intake (never drinker, former drinker, current drinker <3 glasses/wk, current drinker 3–7 glasses/wk, current drinker >7 glasses/wk).

When calcium and magnesium were analyzed separately, we observed overall no association of calcium in drinking water with myocardial infarction or stroke (**Table 4**, Supplemental Table 3). However, for drinking water magnesium and stroke, the highest tertile of drinking water magnesium compared with the lowest was associated with a reduced risk of ischemic stroke (HR: 0.69; 95% CI: 0.54, 0.88), whereas the corresponding HR for hemorrhagic stroke was 0.75 (95% CI: 0.43, 1.31) (**Table 5**). The association of drinking water magnesium with myocardial infarction was not significant (HR: 0.91; 95% CI: 0.70, 1.20 for the highest tertile compared to the lowest; Table 5).

**TABLE 4:** Hazard ratios of myocardial infarction, ischemic stroke, and hemorrhagic stroke by tertiles of drinking water calcium exposure in 26,733 women of the Swedish Mammography Cohort

			HR (95% CI)	
Tertiles of calcium concentration in tap water (mg/L), range (p50)	Cases, *n*	Person-years, *n*	Age-adjusted model^1^	Multivariable adjusted-model^2^
Myocardial infarction				
12.3–32 (29.7)	1071	227,192	1.00	1.00
32.6–37.7 (37.7)	564	143,326	0.82 (0.74, 0.91)	1.01 (0.77, 1.31)
41.2–109.8 (70)	388	84,848	1.00 (0.89, 1.12)	0.93 (0.82, 1.05)
Ischemic stroke				
12.3–32 (29.7)	1219	226,424	1.00	1.00
32.6–37.7 (37.7)	627	143,023	0.79 (0.72, 0.87)	1.17 (0.92, 1.48)
41.2–109.8 (70)	433	84,892	0.98 (0.88, 1.09)	0.95 (0.85, 1.06)
Hemorrhagic stroke				
12.3–32 (29.7)	250	231,788	1.00	1.00
32.6–37.7 (37.7)	125	145,745	0.79 (0.64, 0.98)	1.02 (0.59, 1.76)
41.2–109.8 (70)	77	86,816	0.84 (0.65, 1.09)	0.87 (0.66, 1.13)

1 Adjusted for age (as time scale).

2 Adjusted for age (as time scale), level of education (<9, 9, 10–11, 11–12, >12 y), household income (quartiles), smoking status (never, former <10 cigarettes/d, former ≥10 cigarettes/d, current <10 cigarettes/d, current ≥10 cigarettes/d), BMI (<20, 20–24.9, 25–29.9, and 30 kg/cm^2^), high cholesterol (yes/no), diabetes (yes/no), family history of cardiovascular disease (yes/no), physical activity (walk or bike ≥40 min/wk: yes/no; exercise ≥1 h/wk: yes/no), alcohol intake (never drinker, former drinker, current drinker <3 glasses/wk, current drinker 3–7 glasses/wk, current drinker >7 glasses/wk), and magnesium in drinking water (tertiles).

**TABLE 5:** Hazard ratios of myocardial infarction, ischemic stroke, and hemorrhagic stroke types by tertiles of drinking water magnesium exposure in 26,733 women of the Swedish Mammography Cohort

			HR (95% CI)	
Tertiles of magnesium concentration in tap water (mg/L), range (p50)	Cases, *n*	Person-years, *n*	Age-adjusted model^1^	Multivariable adjusted model^2^
Myocardial infarction				
0.6–4.5 (4.5)	823	186,852	1.00	1.00
4.6–10.5 (7.1)	680	134,219	1.16 (1.05, 1.29)	1.14 (1.02, 1.26)
10.6–13.8 (12.7)	520	134,294	0.86 (0.77, 0.96)	0.91 (0.70, 1.20)
Ischemic stroke				
0.6–4.5 (4.5)	973	186,080	1.00	1.00
4.6–10.5 (7.1)	741	134,175	1.06 (0.97, 1.17)	1.07 (0.96, 1.18)
10.6–13.8 (12.7)	565	134,083	0.78 (0.70, 0.86)	0.69 (0.54, 0.88)
Hemorrhagic stroke				
0.6–4.5 (4.5)	201	190,503	1.00	1.00
4.6–10.5 (7.1)	137	137,303	0.95 (0.76, 1.18)	0.99 (0.79, 1.24)
10.6–13.8 (12.7)	114	136,544	0.78 (0.62, 0.99)	0.75 (0.43, 1.31)

1 Adjusted for age (as time scale).

2 Further adjusted for age (as time scale), level of education (<9, 9, 10–11, 11–12, >12 y), household income (quartiles), smoking status (never, former <10 cigarettes/d, former ≥10 cigarettes/d, current <10 cigarettes/d, current ≥10 cigarettes/d), BMI (<20, 20–24.9, 25–29.9, and 30 kg/cm^2^), high cholesterol (yes/no), diabetes (yes/no), family history of cardiovascular disease (yes/no), physical activity (walk or bike ≥40 min/wk: yes/no; exercise ≥1 h/wk: yes/no), alcohol intake (never drinker, former drinker, current drinker <3 glasses/wk, current drinker 3–7 glasses/wk, current drinker >7 glasses/wk), and calcium in drinking water (tertiles).

In the analyses stratified by median dietary mineral intake, the association of drinking water magnesium with ischemic stroke was stronger among the women with a dietary magnesium intake below the median than among those with an intake above the median (HR: 0.64; 95%, CI: 0.46, 0.89 and HR: 0.76; 95% CI: 0.53, 1.08 for the highest tertile of drinking water magnesium compared with the lowest among those with dietary magnesium intake below and above the median, respectively; **Tables 6** and **7**). Results were similar, although statistically nonsignificant, for myocardial infarction and hemorrhagic stroke (Tables 6 and 7). Drinking water calcium was not associated with any of the outcomes in either stratum (**Tables 8** and **9**). In the analyses according to joint concentrations of magnesium and calcium in drinking water, only high magnesium (i.e., calcium: <50 mg/L; magnesium: ≥10 mg/L) showed a statistically significant inverse association with risk of ischemic and hemorrhagic stroke (HR: 0.81; 95% CI: 0.73, 0.90 and HR: 0.76; 95% CI: 0.60, 0.96, for ischemic and hemorrhagic stroke, respectively; **Supplemental Table 7**).

**TABLE 6:** Hazard ratios of myocardial infarction and stroke by tertiles of drinking water magnesium exposure in 13,207 women with dietary magnesium intake below the median (318 mg/d)

			HR (95% CI)	
Tertiles of magnesium concentration in tap water (mg/L), range (p50)	Number of participants (cases)	Person-years, *n*	Age-adjusted model^1^	Multivariable-adjusted model^2^
Myocardial infarction				
0.6–4.5 (4.5)	5453 (416)	92,028	1.00	1.00
4.6–10.5 (7.1)	3966 (356)	66,481	1.22 (1.06, 1.40)	1.21 (1.04, 1.40)
10.6–13.8 (12.7)	3788 (248)	64,947	0.85 (0.73, 1.00)	0.80 (0.55, 1.15)
Ischemic stroke				
0.6–4.5 (4.5)	5453 (499)	91,724	1.00	1.00
4.6–10.5 (7.1)	3966 (371)	66,454	1.05 (0.92, 1.21)	1.10 (0.95, 1.26)
10.6–13.8 (12.7)	3788 (279)	64,826	0.79 (0.68, 0.91)	0.64 (0.46, 0.89)
Hemorrhagic stroke				
0.6–4.5 (4.5)	5453 (95)	93,970	1.00	1.00
4.6–10.5 (7.1)	3966 (75)	68,042	1.11 (0.82, 1.50)	1.13 (0.82, 1.56)
10.6–13.8 (12.7)	3788 (50)	66,120	0.75 (0.75, 1.06)	0.85 (0.85, 2.03)

1 Adjusted for age (as time scale).

2 Further adjusted for age (as time scale), level of education (< 9, 9, 10–11, 11–12, >12 y), household income (quartiles), smoking status (never, former <10 cigarettes/d, former ≥10 cigarettes/d, current <10 cigarettes/d, current ≥10 cigarettes/d), BMI (<20, 20–24.9, 25–29.9, and 30 kg/cm^2^), high cholesterol (yes/no), diabetes (yes/no), family history of cardiovascular disease (yes/no), physical activity (walk or bike ≥40 min/wk: yes/no; exercise ≥1 h/wk: yes/no), alcohol intake (never drinker, former drinker, current drinker <3 glasses/wk, current drinker 3–7 glasses/wk, current drinker >7 glasses/wk), and calcium in drinking water (tertiles).

**TABLE 7:** Hazard ratios of myocardial infarction and stroke by tertiles of drinking water magnesium exposure in 13,207 women with dietary magnesium intake above the median (318 mg/d)

			HR (95% CI)	
Tertiles of magnesium concentration in tap water (mg/L), range (p50)	Number of participants (cases)	Person-years, *n*	Age-adjusted model^1^	Multivariable adjusted model^2^
Myocardial infarction				
0.6–4.5 (4.5)	5431 (392)	92,997	1.00	1.00
4.6–10.5 (7.1)	3846 (308)	66,316	1.10 (0.95, 1.28)	1.07 (0.91, 1.25)
10.6–13.8 (12.7)	3930 (265)	67,941	0.89 (0.76, 1.04)	1.04 (0.70, 1.54)
Ischemic stroke				
0.6–4.5 (4.5)	5431 (461)	92,511	1.00	1.00
4.6–10.5 (7.1)	3846 (357)	66,263	1.07 (0.93, 1.22)	1.04 (0.90, 1.20)
10.6–13.8 (12.7)	3930 (278)	67,832	0.77 (0.66, 0.89)	0.76 (0.53, 1.08)
Hemorrhagic stroke				
0.6–4.5 (4.5)	5431 (102)	94,632	1.00	1.00
4.6–10.5 (7.1)	3846 (60)	67,747	0.81 (0.59, 1.12)	0.89 (0.63, 1.24)
10.6–13.8 (12.7)	3930 (60)	68,995	0.78 (0.57, 1.07)	0.67 (0.32, 1.39)

1 Adjusted for age (as time scale).

2 Further adjusted for age (as time scale), level of education (<9, 9, 10–11, 11–12, >12 y), household income (quartiles), smoking status (never, former <10 cigarettes/d, former ≥10 cigarettes/d, current <10 cigarettes/d, current ≥10 cigarettes/d), BMI (<20, 20–24.9, 25–29.9, and 30 kg/cm^2^), high cholesterol (yes/no), diabetes (yes/no), family history of cardiovascular disease (yes/no), physical activity (walk or bike ≥40 min/wk: yes/no; exercise ≥1 h/wk: yes/no), alcohol intake (never drinker, former drinker, current drinker <3 glasses/wk, current drinker 3–7 glasses/wk, current drinker >7 glasses/wk), and calcium in drinking water (tertiles).

**TABLE 8:** Hazard ratios of myocardial infarction and stroke by tertiles of drinking water calcium exposure in 13,207 women with dietary calcium intake below the median (1,016 mg/d)

			HR (95% CI)	
Tertiles of calcium concentration in tap water (mg/L), range (p50)	Number of participants (cases)	Person-years, *n*	Age-adjusted model^1^	Multivariable adjusted model^2^
Myocardial infarction				
12.3–32 (29.7)	6464 (512)	111,675	1.00	1.00
32.6–37.7 (37.7)	4162 (257)	71,872	0.79 (0.68, 0.91)	0.99 (0.67, 1.45)
41.2–109.8 (70)	2581 (190)	43,591	0.97 (0.82, 1.15)	0.93 (0.78, 1.10)
Ischemic stroke				
12.3–32 (29.7)	6464 (554)	111,360	1.00	1.00
32.6–37.7 (37.7)	4162 (323)	71,544	0.91 (0.79, 1.05)	1.32 (0.95, 1.84)
41.2–109.8 (70)	2581 (228)	43,382	1.08 (0.93, 1.26)	1.03 (0.88, 1.22)
Hemorrhagic stroke				
12.3–32 (29.7)	6464 (117)	113,799	1.00	1.00
32.6–37.7 (37.7)	4162 (58)	72,982	0.78 (0.57, 1.08)	0.66 (0.27, 1.65)
41.2–109.8 (70)	2581 (34)	44,481	0.76 (0.52, 1.11)	0.80 (0.53, 1.19)

1 Adjusted for age (as time scale).

2 Adjusted for age (as time scale), level of education (<9, 9, 10–11, 11–12, >12 y), household income (quartiles), smoking status (never, former <10 cigarettes/d, former ≥10 cigarettes/d, current <10 cigarettes/d, current ≥10 cigarettes/d), BMI (<20, 20–24.9, 25–29.9, and 30 kg/cm^2^), high cholesterol (yes/no), diabetes (yes/no), family history of cardiovascular disease (yes/no), physical activity (walk or bike ≥40 min/wk: yes/no; exercise ≥1 h/wk: yes/no), alcohol intake (never drinker, former drinker, current drinker <3 glasses/wk, current drinker 3–7 glasses/wk, current drinker >7 glasses/wk), and magnesium in drinking water (tertiles).

**TABLE 9:** Hazard ratios of myocardial infarction and stroke by tertiles of drinking water calcium exposure in 13,207 women with dietary calcium intake above the median (1,016 mg/d)

			HR (95% CI)	
Tertiles of calcium concentration in tap water (mg/L), range (p50)	Number of participants (cases)	Person-years, *n*	Age-adjusted model^1^	Multivariable adjusted model^2^
Myocardial infarction				
12.3–32 (29.7)	6645 (534)	113,162	1.00	1.00
32.6–37.7 (37.7)	4132 (300)	70,011	0.88 (0.76, 1.01)	1.05 (0.72, 1.53)
41.2–109.8 (70)	2430 (192)	40,400	1.05 (0.89, 1.24)	0.95 (0.80, 1.13)
Ischemic stroke				
12.3–32 (29.7)	6645 (648)	112,651	1.00	1.00
32.6–37.7 (37.7)	4132 (295)	70,023	0.69 (0.60, 0.79)	1.01 (0.72, 1.43)
41.2–109.8 (70)	2430 (197)	40,653	0.88 (0.75, 1.03)	0.86 (0.73, 1.02)
Hemorrhagic stroke				
12.3–32 (29.7)	6645 (129)	115,506	1.00	1.00
32.6–37.7 (37.7)	4132 (63)	71,298	0.77 (0.57, 1.04)	1.42 (0.71, 2.81)
41.2–109.8 (70)	2430 (41)	41,441	0.92 (0.65, 1.30)	0.90 (0.63, 1.30)

1 Adjusted for age (as time scale).

2 Adjusted for age (as time scale), level of education (<9, 9, 10–11, 11–12, >12 y), household income (quartiles), smoking status (never, former <10 cigarettes/d, former ≥10 cigarettes/d, current <10 cigarettes/d, current ≥10 cigarettes/d), BMI (<20, 20–24.9, 25–29.9, and 30 kg/cm^2^), high cholesterol (yes/no), diabetes (yes/no), family history of cardiovascular disease (yes/no), physical activity (walk or bike ≥40 min/wk: yes/no; exercise ≥1 h/wk: yes/no), alcohol intake (never drinker, former drinker, current drinker <3 glasses/wk, current drinker 3–7 glasses/wk, current drinker >7 glasses/wk), and magnesium in drinking water (tertiles).

## Discussion

In this large population-based cohort study of postmenopausal women, we observed associations of drinking water with a high concentration of calcium and magnesium with reduced risk of stroke that seemed to be primarily driven by magnesium. Findings for myocardial infarction were less clear. These data suggest that drinking water rich in calcium and magnesium, in particular magnesium, may lower the risk of stroke in postmenopausal women.

For >5 decades, ecological studies have reported on a possible association between drinking water hardness and reduced risk of cardiovascular disease, but the limitations of the ecological study design are well recognized (2). In addition, a number of case-control studies assessed the association of drinking water minerals with various cardiovascular outcomes, mainly death by myocardial infarction, and many (3–8) but not all (19) also found a protective association. Nevertheless, only few cohort studies have been published in this area, and of those, only 1 study (12) could confirm the protective association observed in ecological and case-control studies. In this Finnish study, the authors reported a relative risk reduction of 4.4 (95% CI: 1.3, 25.3) for sudden death due to coronary heart disease at magnesium concentrations comparable (mean 13.1 ± 2.0 mg/L) to those of the high-exposure group in the present study. In other cohort studies, also with comparable or slightly higher drinking water mineral concentrations, and in which adjustments for a large set of potential confounding factors were made, no overall association of drinking water minerals with cardiovascular outcomes was observed (9–11).

Our findings that there is no overall association between drinking water minerals and risk of myocardial infarction accords well with the results of most previous cohort studies (9–11). On the other hand, in our study, drinking water with a high concentration of calcium and magnesium was statistically significantly associated with a lower risk of ischemic and hemorrhagic stroke, and in the separate analyses of the minerals, this association remained for magnesium with ischemic stroke. Only few studies have previously been focusing on stroke. One small prospective cohort study from the Netherlands (9) found inverse but non–statistically significant associations of drinking water magnesium and stroke mortality (HR: 0.77; 95% CI: 0.38, 1.57) at drinking water magnesium concentrations >8.5 mg/L. A second case-control study from Taiwan (7) reported a statistically significant association of drinking water magnesium with death in cerebrovascular disease (OR: 0.75; 95% CI: 0.65, 0.85 and OR: 0.60; 95% CI: 0.52, 0.70 for magnesium concentrations above 7.5 mg/L and 13.5 mg/L, respectively).

There are several biologically plausible mechanisms through which drinking water magnesium may exert cerebroprotective effects. Clinical and experimental evidence suggests that magnesium possess vasodilatory, anti-inflammatory, anti-ischemic, and antiarrhythmic properties (20). Moreover, in a meta-analysis of randomized controlled trials, the authors concluded that magnesium supplementation significantly lowered blood pressure in adults (21), and high blood pressure is a strong risk factor for stroke. In addition, Mendelian randomization studies have found inverse associations of serum magnesium concentration with risk of a multitude of cardiovascular outcomes, including both ischemic and hemorrhagic stroke, further supporting a causal link (22–24). Importantly, such studies found that higher magnesium concentrations are particularly strongly associated with a reduced risk of subarachnoid hemorrhage (24), which was also observed for high calcium and magnesium in our study. This finding might be explained by blood pressure, which is particularly strongly related to this stroke type (25).

A common argument against a possible effect of drinking water magnesium on cardiovascular health is that magnesium intake from drinking water is low in comparison to that from diet and/or supplementation. Nonetheless, the bioavailability of magnesium has been suggested to be higher in drinking water than that in food (26), so it still could make an important contribution to magnesium intake. This notion is further supported by the stronger association of drinking water magnesium with ischemic stroke observed among those with a low dietary magnesium intake. If these results are replicated, they may be used for guiding recommendations on magnesium in drinking water and be an incentive for adding minerals to drinking water with otherwise low concentrations, such as filtered or desalinated drinking water.

This study has several strengths. First, this is one of the largest population-based cohort studies on drinking water calcium and magnesium and cardiovascular disease, with more than 4000 cases of myocardial infarction and stroke. Second, the linkage to several high-quality national registers enabled an almost complete ascertainment of cases, gave us the possibility to trace participants’ migratory patterns over time, and provided us with detailed information on potentially important covariates, such as income and education, which were used as proxies for socioeconomic status. Finally, while tap water has been estimated to account for 78% of all nonalcoholic beverages consumed in Sweden (16), bottled water consumption is very low. [In 2019, Sweden had the lowest estimated bottled water consumption per capita in 25 countries within the European Union (EU), with on average 10 L consumed compared with an EU average of 118 L (15).]

One limitation of our study is the lack of individual information on drinking water consumption. Moreover, the exposure was assigned only based on the concentration at the participants’ residential addresses. Nevertheless, given that most nearby surrounding areas are supplied by the same drinking water treatment plant, any misclassification caused by having different sources at home and at work, for example, is likely small. In addition, a large proportion of the women were already retired at baseline, making this potential issue even smaller. Moreover, our study design limits the possibility to fully disentangle dietary compared with drinking water sources of the minerals as well as their relative proportion. The risk for unmeasured or residual confounding in observational studies is unavoidable, preventing causal inferences based on the results of the study. Nevertheless, we had extensive individual information on major risk factors for myocardial infarction and stroke, socioeconomic variables, and diet, including dietary and supplemental calcium and magnesium intake, and were able to adjust for the most relevant risk factors in the statistical analysis. Last, all participants in our study were female, and given that the overall cardiovascular disease risk in men and women is different, this limits the generalizability of our results.

In conclusion, in this population-based cohort study of postmenopausal women, we observed associations of drinking water with a high concentration of calcium and magnesium, particularly of magnesium, with a reduced risk of ischemic and hemorrhagic stroke. These findings suggest that drinking water with a high magnesium concentration may lower the risk of stroke in postmenopausal women.

## Supplementary Material

nqac186_Supplemental_File

## Data Availability

The data described in the manuscript can be applied for at the SIMPLER homepage: https://www.simpler4health.se/.

## Notes

Supported by grants from Formas, the Swedish Research Council for Environment, Agricultural Sciences and Spatial Planning (2020-01630), and from the Swedish Research Council for Health, Working Life and Welfare (2018-00123). The study sponsors had no role in the initiation of the study or interpretation of the results.

Supplemental Figures 1 and 2 and Supplemental Tables 1–7 are available from the “Supplementary data” link in the online posting of the article and from the same link in the online table of contents at https://academic.oup.com/jn/.

Abbreviations used: DeSo, Demographic statistical areas, EU, European Union; SIMPLER, Swedish Infrastructure for Medical Population–based Life-course and Environmental Research; SMC, Swedish Mammography Cohort.
