# Supplementary material for: Calcium and magnesium in drinking water and risk of myocardial infarction and stroke—a population-based cohort study
Source: Am J Clin Nutr. 2022 Jul 11;116(4):1091–100. doi: 10.1093/ajcn/nqac186 (PMC9535516; doi:10.1093/ajcn/nqac186)
Supplement: nqac186_Supplemental_File [file nqac186_supplemental_file.docx]

On-line Supplementary Material

**Calcium and magnesium in drinking water and risk of myocardial infarction and stroke – a population-based cohort study**

**Emilie Helte^1^, Melle Säve-Söderbergh^1,2^, Susanna C Larsson^1,3^, Agneta Åkesson^1^**

1. Unit of Cardiovascular and Nutritional Epidemiology, Institute of Environmental Medicine, Karolinska Institutet, Stockholm, Sweden.
2. Science Division, Swedish Food Agency, Uppsala, Sweden.
3. Unit of Medical Epidemiology, Department of Surgical Sciences, Uppsala University, Uppsala, Sweden.

**Correspondence:**

Agneta Åkesson

E-mail: [Agneta.Akesson@ki.se](mailto:Agneta.Akesson@ki.se)

**Supplementary Table 1**

Baseline age-standardized main characteristics of the study population (n=26,733) by tertiles of drinking water calcium distribution.

| **Tertile of drinking water calcium concentration,** mg/L mean ± SD | **Tertile 1**  27 ± 6 | **Tertile 2**  38 ± 0.6 | **Tertile 3**  72 ± 15 |
| --- | --- | --- | --- |
| N participants | 13,269 | 8,389 | 5,075 |
| Age, years, mean (±SD) | 62 (± 9) | 62 (± 10) | 61 (± 9) |
| Education, years, % |  |  |  |
| ≤ 9 | 45 | 30 | 46 |
| 10 – 11 | 30 | 25 | 32 |
| ≥ 12 | 24 | 44 | 23 |
| Household income, 1000 SEK^1^/year, mean (±SD) | 232 (± 137) | 251 (± 176) | 228 (± 219) |
| Median area level income, 1000 SEK^1^/year, mean (±SD) | 240 (± 36) | 261 (± 46) | 239 (± 31) |
| Smoking status, % |  |  |  |
| Never | 53 | 51 | 56 |
| Former, < 10 cigarettes/day | 12 | 13 | 10 |
| Former, ≥ 10 cigarettes/day | 11 | 12 | 11 |
| Current, < 10 cigarettes/day | 10 | 12 | 10 |
| Current, ≥ 10 cigarettes/day | 14 | 12 | 13 |
| BMI, kg/cm^2^, % | 25.0 (± 3.9) | 24.5 (± 3.9) | 25.2 (± 4.0) |
| High cholesterol, % | 6 | 9 | 8 |
| Prevalent diabetes, % | 4 | 4 | 5 |
| Family history of cardiovascular disease, % | 17 | 16 | 17 |
| Physical activity, % |  |  |  |
| Walk/bike ≥ 40 minutes day | 36 | 41 | 35 |
| Exercise ≥ 1 hour week | 82 | 80 | 82 |
| Alcohol consumption, % |  |  |  |
| Never drinker | 12 | 9 | 14 |
| Former drinker | 4 | 3 | 5 |
| Drinker, ≤ 3 glasses/week | 54 | 52 | 54 |
| Drinker, 3-7 glasses/week | 21 | 26 | 20 |
| Drinker, 7 ≥ glasses/week | 8 | 11 | 7 |
| Calcium intake, mg/day, mean (±SD) | 1,050 (± 301) | 1,048 (± 301) | 1,038 (± 303) |
| Magnesium intake,  mg/day, mean (±SD) | 319 (± 43) | 321 (± 44) | 319 (± 42) |
| Calcium supplements, % | 1 | 1 | 1 |
| Magnesium supplements, % | 4 | 4 | 3 |

Abbreviations: SD = standard deviation, BMI = body mass index,

^1^1000 SEK = 100 EUR or 121 USD (exchange rate February 2021)

**Supplementary Table 2**

Baseline age-standardized main characteristics of the study population (n=26,733) by tertiles of drinking water magnesium distribution.

| **Tertile of drinking water magnesium concentration** mg/L mean ± SD | **Tertile 1**  4 ± 0.5 | **Tertile 2**  7 ± 2 | **Tertile 3**  13 ± 0.2 |
| --- | --- | --- | --- |
| N participants | 11,013 | 7,913 | 7,807 |
| Age, years, mean (±SD) | 62 (± 9) | 62 (± 9) | 62 (± 9) |
| Education, years, % |  |  |  |
| ≤ 9 | 44 | 48 | 29 |
| 10 – 11 | 30 | 31 | 25 |
| ≥ 12 | 26 | 21 | 46 |
| Household income, 1000 SEK^1^/year, mean (±SD) | 235 (± 178) | 225 (± 146) | 251 (± 174) |
| Median area level income, 1000 SEK^1^/year, mean (±SD) | 242 (± 38) | 237 (± 32) | 262 (± 46) |
| Smoking status, % |  |  |  |
| Never | 54 | 54 | 51 |
| Former, < 10 cigarettes/day | 11 | 11 | 13 |
| Former ≥ 10 cigarettes/day | 11 | 11 | 12 |
| Current < 10 cigarettes/day | 10 | 10 | 12 |
| Current ≥ 10 cigarettes/day | 13 | 14 | 12 |
| BMI, kg/cm^2^, % | 25.0 (± 3.9) | 25.2 (± 4.0) | 24.4 (± 3.8) |
| High cholesterol, % | 7 | 7 | 9 |
| Prevalent diabetes, % | 4 | 5 | 4 |
| Family history of cardiovascular disease, % | 17 | 18 | 16 |
| Physical activity, % |  |  |  |
| Walk/bike ≥ 40 minutes day | 36 | 35 | 42 |
| Exercise ≥ 1 hour week | 82 | 82 | 80 |
| Alcohol consumption, % |  |  |  |
| Never drinker | 12 | 14 | 8 |
| Former drinker | 4 | 5 | 3 |
| Drinker, ≤ 3 glasses/week | 53 | 55 | 51 |
| Drinker, 3-7 glasses/week | 22 | 20 | 26 |
| Drinker, 7 ≥ glasses/week | 9 | 7 | 11 |
| Calcium intake, mg/day, mean (±SD) | 1,056 (± 302) | 1,033 (± 301) | 1,050 (± 301) |
| Magnesium intake, mg/day, mean (±SD) | 320 (± 43) | 319 (± 43) | 321 (± 45) |
| Calcium supplements, % | 1 | 1 | 1 |
| Magnesium supplements, % | 4 | 4 | 4 |

Abbreviations: SD = standard deviation, BMI = body mass index,

^1^1000 SEK = 100 EUR or 121 USD (exchange rate February 2021)

**Supplementary Table 3**

Hazard ratios of myocardial infarction, including coronary revascularization procedures, by drinking water calcium and magnesium exposure in 26,733 women of The Swedish Mammography Cohort.

| **Exposure** | **Cases (n)** | **Person  years (n)** | **Model 1^2^ HR (95%CI)** | **Model 2^3^ HR (95%CI)** | **Model 3^4^ HR (95%CI)** | **Model 4^5^ HR (95%CI)** |
| --- | --- | --- | --- | --- | --- | --- |
| **Drinking water calcium and magnesium**^1^ | | | | | | |
| Ca/Mg < 50/10 mg/L | 1,313 | 247,087 | 1.00 | 1.00 | 1.00 | 1.00 |
| Ca/Mg ≥ 50/10 mg/L | 968 | 205,529 | 0.89 (0.82-0.97) | 0.93 (0.86-1.01) | 0.94 (0.87-1.03) | 0.93 (0.85-1.01) |
| **Drinking water calcium (mg/L)** | | | | | | |
| 12.3 – 32 (29.7) | 1,211 | 225,675 | 1.00 | 1.00 | 1.00 | 1.00 |
| 32.6 – 37.7 (37.7) | 623 | 142,738 | 0.81 (0.73-0.89) | 0.97 (0.76-1.25) | 0.98 (0.76-1.26) | 0.97 (0.75-1.25) |
| 41.2 – 109.8 (70) | 447 | 84,203 | 1.02 (0.91-1.14) | 0.94 (0.84-1.05) | 0.94 (0.84-1.06) | 0.94 (0.84-1.05) |
| **Drinking water magnesium (mg/L)** | | | | | | |
| 0.6 – 4.5 (4.5) | 933 | 185,726 | 1.00 | 1.00 | 1.00 | 1.00 |
| 4.6 – 10.5 (7.1) | 773 | 113,137 | 1.17 (1.06-1.29) | 1.13 (1.03-1.25) | 1.13 (1.02-1.25) | 1.13 (1.03-1.25) |
| 10.6 – 13.8 (12.7) | 575 | 133,753 | 0.85 (0.76-0.94) | 0.93 (0.72-1.20) | 0.94 (0.72-1.21) | 0.93 (0.72-1.20) |

Abbreviations: Ca = calcium, Mg = magnesium, HR = hazard ratio, CI = confidence interval

^1^Low calcium and magnesium is defined as Ca <50 mg/L and Mg <10 mg/L, and high calcium and magnesium is defined as Ca ≥50 mg/L or Mg ≥10 mg/L

^2^Adjusted for age (as timescale)

^3^Further adjusted for level of education (< 9 yrs, 9 yrs, 10-11 yrs, 11-12 yrs, >12 yrs), household income (quartiles), smoking status (never, former < 10 cig/day, former ≥ 10 cig/day current < 10 cig/day, current ≥ 10 cig/day), body mass index (<20, 20-24.9, 25-29.9 and 30 kg/cm^2^), high cholesterol (yes/no), diabetes (yes/no), family history of cardiovascular disease (yes/no), physical activity (walk or bike ≥ 40 min/week: yes/no; exercise ≥ 1 hour/week: yes/no) and alcohol intake (never drinker, former drinker, current drinker < 3 glasses/week, current drinker 3-7 glasses/week, current drinker > 7 glasses/week). In the separate analyses of calcium and magnesium, models are also adjusted for the drinking water concentration of the mineral that is not under evaluation (tertiles).

^4^Model 2 further adjusted for median income in the area of residence (continuous)

^5^Model 2 further adjusted for dietary calcium (continuous), dietary magnesium (continuous), calcium supplements (yes/no) and magnesium supplements (yes/no). In the separate analyses of calcium and magnesium, adjustments for intake from diet and supplements were made only for the mineral under evaluation.

**Supplementary Table 4**

Hazard ratios of myocardial infarction and stroke by drinking water calcium and magnesium exposure in 26,733 women of The Swedish Mammography Cohort. Results from models additionally adjusted for dietary intake of calcium and use of calcium supplements or median area level income.

| **Drinking water calcium and magnesium exposure**^1^ | **Cases (n)** | **Person  years (n)** | **Hazard ratio (95% CI)** | | |
| --- | --- | --- | --- | --- | --- |
|  |  |  | **Age adjusted model^2^** | **Multivariable adjusted model 1^3^** | **Multivariable adjusted model 2^4^** |
| **Myocardial Infarction** |  |  |  |  |  |
| Ca/Mg < 50/10 mg/L | 1,163 | 248,693 | 1.00 | 1.00 | 1.00 |
| Ca/Mg ≥ 50/10 mg/L | 860 | 206,671 | 0.89 (0.81 - 0.97) | 0.93 (0.85 - 1.01) | 0.94 (0.86 - 1.03) |
| **Ischemic stroke** |  |  |  |  |  |
| Ca/Mg < 50/10 mg/L | 1,326 | 247,877 | 1.00 | 1.00 | 1.00 |
| Ca/Mg ≥ 50/10 mg/L | 953 | 206,462 | 0.85 (0.79 - 0.93) | 0.87 (0.80 - 0.95) | 0.89 (0.81 - 0.97) |
| **Composite hemorrhagic stroke** | |  |  |  |  |
| Ca/Mg < 50/10 mg/L | 274 | 253,631 | 1.00 | 1.00 | 1.00 |
| Ca/Mg ≥ 50/10 mg/L | 178 | 210,718 | 0.79 (0.65 - 0.95) | 0.78 (0.65 - 0.95) | 0.79 (0.65 - 0.96) |
| **Intracerebral hemorrhage** |  |  |  |  |  |
| Ca/Mg < 50/10 mg/L | 207 | 254,014 | 1.00 | 1.00 | 1.00 |
| Ca/Mg ≥ 50/10 mg/L | 142 | 210,903 | 0.83 (0.67 - 1.03) | 0.83 (0.66 - 1.03) | 0.82 (0.66 - 1.03) |
| **Subarachnoid hemorrhage** |  |  |  |  |  |
| Ca/Mg < 50/10 mg/L | 75 | 254,473 | 1.00 | 1.00 | 1.00 |
| Ca/Mg ≥ 50/10 mg/L | 41 | 211,190 | 0.67 (0.45 - 0.97) | 0.67 (0.45 - 0.98) | 0.69 (0.47-1.02) |

Abbreviations: Ca = calcium, Mg = magnesium, HR = hazard ratio, CI = confidence interval

^a^Low calcium and magnesium is defined as Ca <50 mg/L and Mg <10 mg/L, and high calcium and magnesium is defined as Ca ≥50 mg/L or Mg ≥10 mg/L

^2^Adjusted for age (as timescale)

^3^Adjusted for level of education (< 9 yrs, 9 yrs, 10-11 yrs, 11-12 yrs, >12 yrs), household income (quartiles), smoking status (never, former < 10 cig/day, former ≥ 10 cig/day current < 10 cig/day, current ≥ 10 cig/day), body mass index (<20, 20-24.9, 25-29.9 and 30 kg/cm^2^), high cholesterol (yes/no), diabetes (yes/no), family history of cardiovascular disease (yes/no), physical activity (walk or bike ≥ 40 min/week: yes/no; exercise ≥ 1 hour/week: yes/no), alcohol intake (never drinker, former drinker, current drinker < 3 glasses/week, current drinker 3-7 glasses/week, current drinker > 7 glasses/week), dietary intake of calcium (continuous), dietary intake of magnesium (continuous), use of calcium supplements (yes/no) and use of magnesium supplements (yes/no) .

^4^Adjusted for level of education (< 9 yrs, 9 yrs, 10-11 yrs, 11-12 yrs, >12 yrs), household income (quartiles), smoking status (never, former < 10 cig/day, former ≥ 10 cig/day current < 10 cig/day, current ≥ 10 cig/day), body mass index (<20, 20-24.9, 25-29.9 and 30 kg/cm^2^), high cholesterol (yes/no), diabetes (yes/no), family history of cardiovascular disease (yes/no), physical activity (walk or bike ≥ 40 min/week: yes/no; exercise ≥ 1 hour/week: yes/no), alcohol intake (never drinker, former drinker, current drinker < 3 glasses/week, current drinker 3-7 glasses/week, current drinker > 7 glasses/week) and median income in the area of residence (continuous)

**Supplementary Table 5**

Hazard ratios of myocardial infarction, and stroke by tertiles of drinking water calcium exposure in 26,733 women of The Swedish Mammography Cohort. Results from models additionally adjusted for dietary intake of magnesium and use of magnesium supplements or median area level income.

| **Tertiles of calcium concentration in tap water (mg/L), range (p50)** | **Cases (n)** | **Person  years (n)** | **Hazard ratio (95% confidence interval)** | | |
| --- | --- | --- | --- | --- | --- |
|  |  |  | **Age adjusted model^1^** | **Multivariable adjusted model 1^2^** | **Multivariable adjusted model 2^3^** |
| **Myocardial Infarction** |  |  |  |  |  |
| 12.3 – 32 (29.7) | 1,071 | 227,192 | 1.00 | 1.00 | 1.00 |
| 32.6 – 37.7 (37.7) | 564 | 143,326 | 0.82 (0.74 - 0.91) | 1.00 (0.77 - 1.31) | 1.02 (0.78-1.33) |
| 41.2 – 109.8 (70) | 388 | 84,848 | 1.00 (0.89 - 1.12) | 0.93 (0.82 - 1.04) | 0.93 (0.82-1.05) |
| **Ischemic stroke** |  |  |  |  |  |
| 12.3 – 32 (29.7) | 1,219 | 226,424 | 1.00 | 1.00 | 1.00 |
| 32.6 – 37.7 (37.7) | 627 | 143,023 | 0.79 (0.72 - 0.87) | 1.17 (0.92 - 1.48) | 1.18 (0.93-1.49) |
| 41.2 – 109.8 (70) | 433 | 84,892 | 0.98 (0.88 - 1.09) | 0.95 (0.85 - 1.06) | 0.95 (0.85-1.06) |
| **Hemorrhagic stroke** |  |  |  |  |  |
| 12.3 – 32 (29.7) | 250 | 231,788 | 1.00 | 1.00 | 1.00 |
| 32.6 – 37.7 (37.7) | 125 | 145,745 | 0.79 (0.64 - 0.98) | 1.02 (0.59 – 1.75) | 1.02 (0.59 – 1.77) |
| 41.2 – 109.8 (70) | 77 | 86,816 | 0.84 (0.65 - 1.09) | 0.86 (0.66 - 1.13) | 0.87 (0.66 - 1.13) |

^1^Adjusted for age (as timescale)

^2^Adjusted for age (as timescale, level of education (< 9 yrs, 9 yrs, 10-11 yrs, 11-12 yrs, >12 yrs), household income (quartiles), smoking status (never, former < 10 cig/day, former ≥ 10 cig/day current < 10 cig/day, current ≥ 10 cig/day), body mass index (<20, 20-24.9, 25-29.9 and 30 kg/cm^2^), high cholesterol (yes/no), diabetes (yes/no), family history of cardiovascular disease (yes/no), physical activity (walk or bike ≥ 40 min/week: yes/no; exercise ≥ 1 hour/week: yes/no), alcohol intake (never drinker, former drinker, current drinker < 3 glasses/week, current drinker 3-7 glasses/week, current drinker > 7 glasses/week), magnesium in drinking water (tertiles), dietary intake of calcium (continuous) and use of calcium supplements (yes/no).

^3^Adjusted for level of education (< 9 yrs, 9 yrs, 10-11 yrs, 11-12 yrs, >12 yrs), household income (quartiles), smoking status (never, former < 10 cig/day, former ≥ 10 cig/day current < 10 cig/day, current ≥ 10 cig/day), body mass index (<20, 20-24.9, 25-29.9 and 30 kg/cm^2^), high cholesterol (yes/no), diabetes (yes/no), family history of cardiovascular disease (yes/no), physical activity (walk or bike ≥ 40 min/week: yes/no; exercise ≥ 1 hour/week: yes/no), alcohol intake (never drinker, former drinker, current drinker < 3 glasses/week, current drinker 3-7 glasses/week, current drinker > 7 glasses/week), magnesium in drinking water (tertiles) and median income in the area of residence (continuous).

**Supplementary Table 6**

Hazard ratios of myocardial infarction and stroke by tertiles of drinking water magnesium exposure in 26,733 women of The Swedish Mammography Cohort.

| **Tertiles of magnesium concentration in tap water (mg/L), range (p50)** | **Cases (n)** | | **Person  years (n)** | **Hazard ratio (95% confidence interval)** | | |
| --- | --- | --- | --- | --- | --- | --- |
|  |  |  |  | **Age adjusted model^1^** | **Multivariable adjusted model 1^2^** | **Multivariable adjusted model 2^3^** |
| **Myocardial Infarction** | |  |  |  |  |  |
| 0.6 – 4.5 (4.5) | | 823 | 186,852 | 1.00 | 1.00 | 1.00 |
| 4.6 – 10.5 (7.1) | | 680 | 134,219 | 1.16 (1.05 - 1.29) | 1.14 (1.02 - 1.26) | 1.13 (1.02 - 1.26) |
| 10.6 – 13.8 (12.7) | | 520 | 134,294 | 0.86 (0.77 - 0.96) | 0.91 (0.70 - 1.20) | 0.92 (0.70 - 1.21) |
| **Ischemic stroke** | |  |  |  |  |  |
| 0.6 – 4.5 (4.5) | | 973 | 186,080 | 1.00 | 1.00 | 1.00 |
| 4.6 – 10.5 (7.1) | | 741 | 134,175 | 1.06 (0.97 – 1.17) | 1.07 (0.96 – 1.18) | 1.06 (0.96 - 1.18) |
| 10.6 – 13.8 (12.7) | | 565 | 134,083 | 0.78 (0.70 – 0.86) | 0.69 (0.54 – 0.88) | 0.69 (0.55 - 0.88) |
| **Hemorrhagic stroke** | |  |  |  |  |  |
| 0.6 – 4.5 (4.5) | | 201 | 190,503 | 1.00 | 1.00 | 1.00 |
| 4.6 – 10.5 (7.1) | | 137 | 137,303 | 0.95 (0.76 – 1.18) | 0.99 (0.79 – 1.24) | 0.99 (0.78 - 1.24) |
| 10.6 – 13.8 (12.7) | | 114 | 136,544 | 0.78 (0.62 – 0.99) | 0.75 (0.43 – 1.31) | 0.75 (0.43 -1.31) |

^1^Adjusted for age (as timescale)

^2^Adjusted for age (as timescale), level of education (< 9 yrs, 9 yrs, 10-11 yrs, 11-12 yrs, >12 yrs), household income (quartiles), smoking status (never, former < 10 cig/day, former ≥ 10 cig/day current < 10 cig/day, current ≥ 10 cig/day), body mass index (<20, 20-24.9, 25-29.9 and 30 kg/cm^2^), high cholesterol (yes/no), diabetes (yes/no), family history of cardiovascular disease (yes/no), physical activity (walk or bike ≥ 40 min/week: yes/no; exercise ≥ 1 hour/week: yes/no), alcohol intake (never drinker, former drinker, current drinker < 3 glasses/week, current drinker 3-7 glasses/week, current drinker > 7 glasses/week), calcium in drinking water (tertiles), dietary intake of magnesium (continuous) and use of magnesium supplements (yes/no).

^3^Adjusted for age (as timescale), level of education (< 9 yrs, 9 yrs, 10-11 yrs, 11-12 yrs, >12 yrs), household income (quartiles), smoking status (never, former < 10 cig/day, former ≥ 10 cig/day current < 10 cig/day, current ≥ 10 cig/day), body mass index (<20, 20-24.9, 25-29.9 and 30 kg/cm^2^), high cholesterol (yes/no), diabetes (yes/no), family history of cardiovascular disease (yes/no), physical activity (walk or bike ≥ 40 min/week: yes/no; exercise ≥ 1 hour/week: yes/no), alcohol intake (never drinker, former drinker, current drinker < 3 glasses/week, current drinker 3-7 glasses/week, current drinker > 7 glasses/week), calcium in drinking water (tertiles) and median income in the area of residence (continuous).

**Supplementary Table 7**

Hazard ratios of myocardial infarction, ischemic and hemorrhagic stroke by categories of drinking water calcium and magnesium exposure in 24,969^1^ women of The Swedish Mammography Cohort.

| **Drinking water calcium and magnesium exposure** | **Cases (n)** | **Person  years (n)** | **Hazard ratio (95% confidence interval)** | |
| --- | --- | --- | --- | --- |
|  |  |  | **Age adjusted model^2^** | **Multivariable adjusted model^3^** |
| **Myocardial Infarction** |  |  |  |  |
| Ca/Mg < 50/10 mg/L | 1,163 | 248,693 | 1.00 | 1.00 |
| Ca ≥ 50 mg/L & Mg <10 mg/L | 211 | 40,068 | 1.02 (0.88-1.19) | 0.98 (0.85-1.14) |
| Ca <50 mg/L & Mg ≥10 mg/L | 511 | 131,492 | 0.81 (0.73–0.90) | 0.91 (0.81-1.01) |
| **Ischemic stroke** |  |  |  |  |
| Ca/Mg < 50/10 mg/L | 1,326 | 247,877 | 1.00 | 1.00 |
| Ca ≥ 50 mg/L & Mg <10 mg/L | 226 | 46,145 | 0.96 (0.83-1.10) | 0.94 (0.82-1.09) |
| Ca <50 mg/L & Mg ≥10 mg/L | 558 | 131,259 | 0.76 (0.69–0.84) | 0.81 (0.73–0.90) |
| **Hemorrhagic stroke** |  |  |  |  |
| Ca/Mg < 50/10 mg/L | 274 | 253,631 | 1.00 | 1.00 |
| Ca ≥ 50 mg/L & Mg <10 mg/L | 36 | 47,178 | 0.73 (0.52-1.04) | 0.75 (0.53-1.07) |
| Ca <50 mg/L & Mg ≥10 mg/L | 112 | 133,683 | 0.77 (0.62–0.96) | 0.76 (0.60–0.96) |

Abbreviations: Ca = calcium, Mg = magnesium

^1^Excluding 1,764 women with drinking water high in both Ca and Mg

^2^Adjusted for age (as timescale)

^3^Adjusted for age (as timescale), level of education (< 9 yrs, 9 yrs, 10–11 yrs, 11–12 yrs, >12 yrs), household income (quartiles), area level income (quartiles), smoking status (never, former < 10 cig/day, former ≥ 10 cig/day current < 10 cig/day, current ≥ 10 cig/day), body mass index (<20, 20–24.9, 25–29.9 and 30 kg/cm^2^), high cholesterol (yes/no), diabetes (yes/no), family history of cardiovascular disease (yes/no), physical activity (walk or bike ≥ 40 min/week: yes/no; exercise ≥ 1 hour/week: yes/no) and alcohol intake (never drinker, former drinker, current drinker < 3 glasses/week, current drinker 3–7 glasses/week, current drinker > 7 glasses/week).


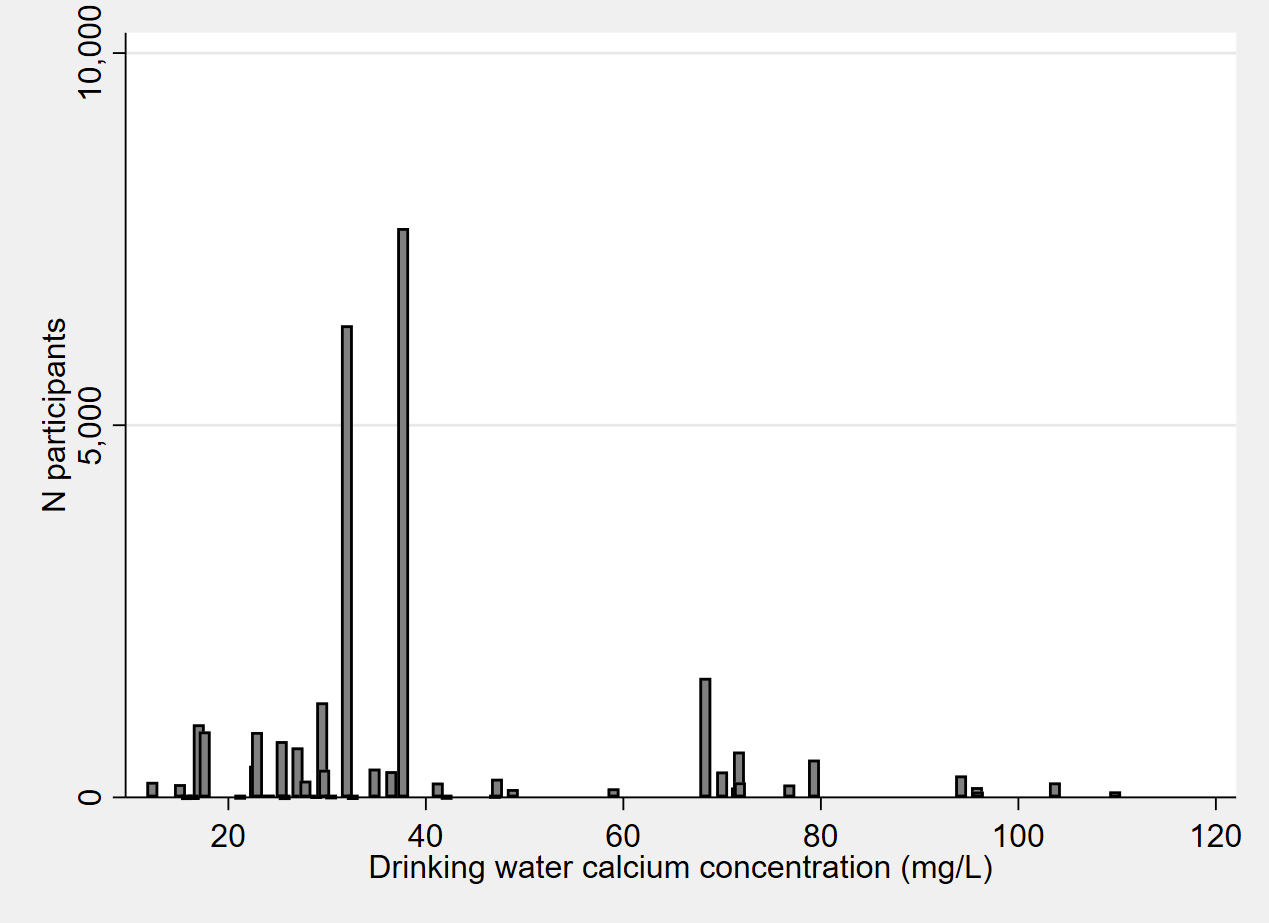
 **Supplementary Figure 1.** Distribution of drinking water calcium concentrations in the study area


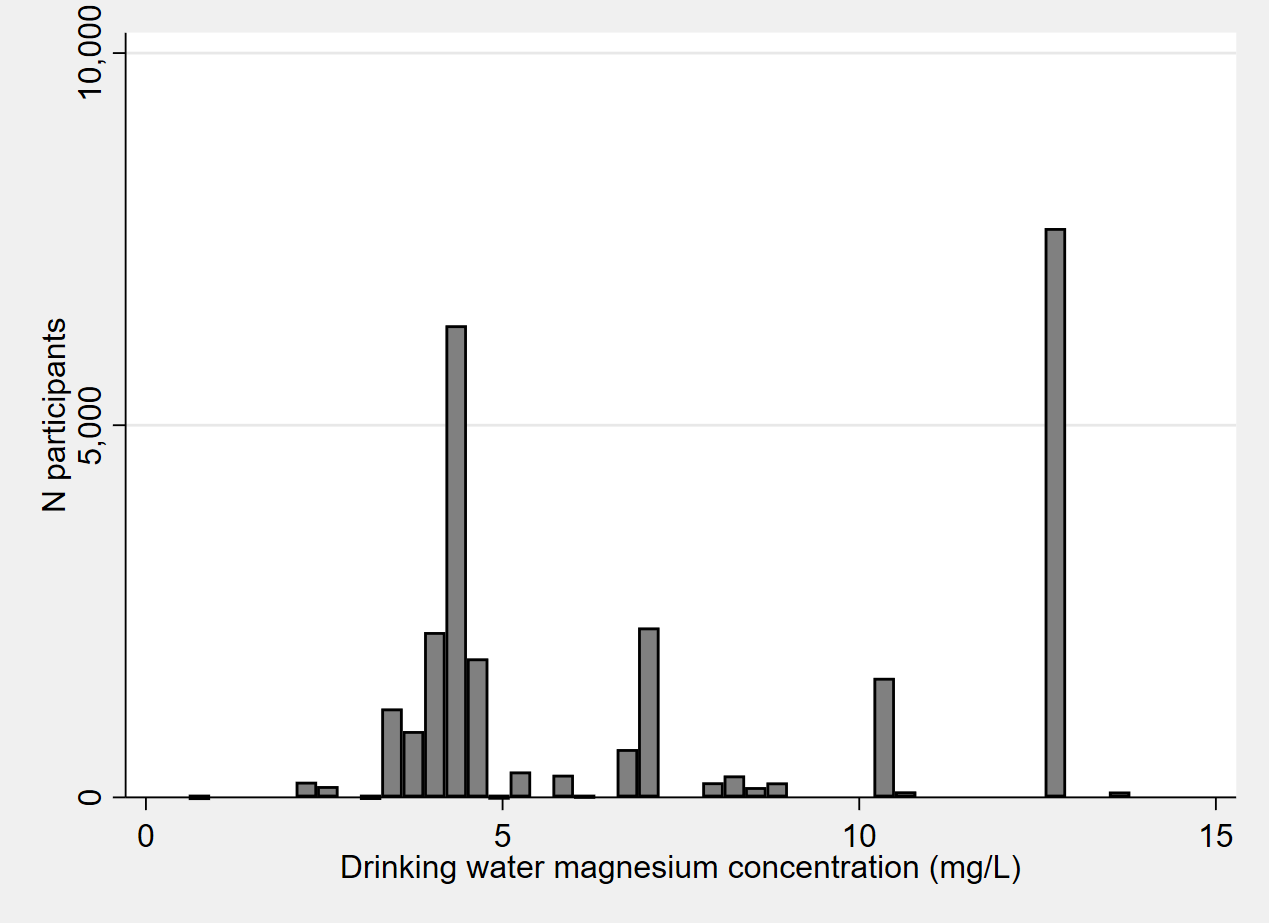
 **Supplementary Figure 2.** Distribution of drinking water magnesium concentrations in the study area
